# Progress in Surgery

**Published:** 1896-05-23

**Authors:** 


### Progress in Surgery.

### RESPIRATORY SURGERY.

**Foreign Bodies in the Bronchial Tubes.**—Godlee,[1] in a paper read at the Royal Medico-Chirurgical Society, describes the effects produced by the retention of foreign bodies in the bronchial tubes for lengthened periods. Five cases were recorded, and in all bronchiectasis of one lung had resulted from the impaction of the foreign body in its bronchial tube. It was pointed out that in all cases of unilateral dilatation of the bronchial tubes, inquiry should be made as to the inhalation of any foreign body. In some of the cases bronchiectatic cavities were opened. Dr. Kenneth McLeod referred to a case where phthisis had been diagnosed, but the necropsy showed a piece of tin lodged in the bronchus with general bronchiectasis of the lung. The boy was demented, and hence no history could be obtained. Dr. Fowler considers that the bronchiectasis is usually of the fusiform variety, and that the symptoms generally follow a definite course, beginning with cough and expectoration, the latter becoming fœtid; then pleurisy and progressive emaciation set in, and, later still, diffuse broncho-pneumonia followed by gangrene. It could generally be taken for granted, he thought, that if there was no definite history of pleurisy, then there would be found no pleural adhesions.

**Sub-diaphragmatic Abscess.**—Mr. Godlee discusses this in a clinical lecture.[2] If the collection of pus is intra-peritoneal, he thinks it will have arisen from a perforation of the stomach, duodenum, or colon, or from disease of the liver or spleen; whereas, if extra-peritoneal, it may start from such structures as the kidneys, the posterior part of the liver where it is uncovered by peritoneum, the cæcum, and the vertebræ and ribs. He draws attention to the fact that abscesses, either in the liver or between it and the diaphragm, may cause dulness reaching as high up as the second rib, and records a case of suppurating hydatid cyst of the liver, which was opened in mistake for an empyæma, in which the dulness ascended to this level. The importance of recognising this is pointed out, as otherwise the healthy pleural cavity may be opened, in the attempt to evacuate the pus. The symptoms of what has been called subphrenic pyo-pneumothorax are described. This condition is due to perforation of the stomach or duodenum, gas and pus collecting between the diaphragm and the liver shut in by peritoneal adhesions. The conditions may be mistaken for true pyo-pneumothorax, and by incising the chest through an intercostal space the healthy pleura infected, whereas an incision below the ribs in front would safely open the abscess-cavity. The distinction between the two conditions depends on the history of the case and the presence of abdominal swelling. One very interesting case of the kind is recorded in which there was a serous pleural effusion, above the collection of pus and gas beneath the diaphragm. First stinking pus was drawn off through the ninth interspace in the scapular line, then serous fluid at the same spot. Finally the abscess was opened low down over the side of the chest, the healthy pleura being reflected from the diaphragm before the latter was incised. An interesting case of sub-diaphragmatic abscess is also recorded, due to caries of the dorsal spine. The dulness reached as high on the left side as the eighth rib behind, and resonance was impaired to the angle of the scapula, the heart being pushed over to the right, and a loud systolic displacement bruit produced. The murmur was very distinctly heard all over the region occupied by the abscess, but was not heard at the apex of the heart.

Dr. Carl Beck[3] considers that the diagnosis of subphrenic pyo-pneumothorax can be made by observing that instead of liver dulness, a profound and full sound is present, and below the right costal arch the liver is pushed far into the abdomen, and its lower border is easily recognised by palpation and percussion. He thinks the history is often an important guide as to the location of the abscess. In subphrenic abscess there is often a history of previous abdominal disturbance, but none of cough and expectoration. In encysted pleuritic effusion the distinction from subphrenic abscess is often impossible. Spontaneous cure in subphrenic abscess he considers extremely rare—only six times in 104 cases. He opens the abscess by resecting a portion of the eighth, ninth, or tenth rib in the mid-axillary line, and does not try and avoid the pleura, as he thinks the danger of pneumothorax is largely prevented by loss of the "aspirating" power of the diaphragm from the interference with its action by the subphrenic abscess. He does not fear infection of the healthy pleura by the pus. In one case which he records, in which the pleural sac was encountered in the operation, and the subphrenic abscess opened through it, no pneumothorax occurred, and the patient made an uninterrupted recovery; whilst in another the air entered the pleura with considerable noise, and the face of the patient assumed a cadaveric appearance, and the pulse entirely disappeared. After the cavity had been packed with iodoform gauze, and stimulants administered, the patient rallied, and on the following day the abscess was opened, the edges of the diaphragmatic incision being sewn to the skin.

Dr. Leith records[4] a case of empyæma due to perforation of a gastric ulcer in a child aged ten. An adhesion had first taken place between the stomach and spleen at the site of the ulcer, and he thinks "some form of pyogenic organisms had made its way from the stomach through the floor of the ulcer into the adhesions, and thus gradually caused the suppuration track, which culminated in the necrosis of the diaphragm, and by an extension into the pleura set up the empyæma." There were no symptoms of gastric ulcer. On opening the empyæma very fetid pus escaped. Reference is made to a case of gastric ulcer in a youth of nineteen, which penetrated into the left ventricle of the heart. The rarity of gastric ulcer occurring in children is pointed out. Empyæma is described as arising from perforations of a gastric ulcer either by the stomach wall becoming adherent to the diaphragm, and the ulcer perforating through its coats and then through the diaphragm, or by the formation of a more or less perfectly circumscribed peritonitis, which gives rise to an abdominal abscess, bounded above by the diaphragm. The pus may then discharge into the pleural cavity, or septic organisms may be carried from the abscess into the pleura by means of the lymphatics. The diagnosis of perforation of a gastric ulcer as the cause of empyæma can be made only if the symptoms of gastric ulcer have been marked, and then only with certainty if, as sometimes happens, portions of food are found in the pus or sputum. Empyæma from perforation of a gastric ulcer is nearly always on the left side.

**Abscess of the Lung.**—Quince[5] has operated on 17 cases, and has collected the records of 34 more, making a total of 51. Many more patients recover after operation in acute cases than in chronic. He estimates that complete success will be attained after operation in two out of every three cases. If on operating he finds the pleural surfaces non-adherent, he applies caustics until adhesions have taken place and then opens the abscess.

A case in which an abscess of the lung secondary to pneumonia was successfully drained is recorded[6] by P. Walton. Although the symptoms had existed for more than a year, the pleural surfaces were not adherent and had to be stitched together. The lung was divided by the thermo-cautery used at a dull red heat. He advocates free incision with extensive rib resection, and the use of the thermo-cautery instead of the knife to divide the lung.

**A Cervical Rib**, which interfered with the patient's work as a soldier, was removed by F. de Quervain.[7] The pleura was wounded, but a gauze plug, immediately applied, prevented pneumothorax. Six previous cases are recorded.

[1] Lancet, March 28, 1896. [2] International Clinics, IV., 3. [3] Medical Record, Feb. 15, 1896. [4] International Clinics, IV. 4. [5] Mitteilungen aus den Greizbieten der Medizin und Chirurgie, Bd. 1, Hft. 1. Epitome B.M.J., April 11, 1896. [6] La Belg. Méd., Nov. 7, 1895; Epitome B.M.J., Jan, 11, 1896. [7] Central für Chir., No. 4[illegible]7, 1895, and Quarterly Med. J., Jan., 1896.

### DISEASES OF THE UPPER AIR PASSAGES.

(*Continued from page* 112.)

**Rhinitis.**—Diseases of the mouth, nose, and throat are discussed as etiological factors in chronic glandular gastritis by Fenton Turck,[24] of Chicago. He states that the percentage of HCl in the stomach alone is not a factor in preventing the development of micro-organisms, as formerly supposed, since micro-organisms are found present in great numbers where there has been an excess of HCl running as high as 0·3 per cent. and 0·35 per cent. He refers to Miller's observations of mouth bacteria and his experiments upon the invasion from the mouth, including the digestive tract; even the staphylococcus and streptococcus have been found growing in the mouth, and Biondi found five pyogenic developing there. Miller demonstrated in four of the pyogenic micro-organisms their power of exciting inflammation, and cites Kaczarowksi, who held that the infectious process in inflammation of the gums was caused by micro-organisms, for disinfection of inflamed gums or oral cavity in teething children stops both the inflammation and also the concomitant catarrh of the mucous membrane of the respiratory and digestive tracts. Turck, from his own experiments, observes that all micro-organisms that excite inflammation are not necessarily pyogenic; even lactic acid micro-organisms form toxines that cause "catarrhal inflammation." After a series of careful investigations, he formed the conclusions: (1) That clinical observation in many cases indicates a marked relation between diseases of the mouth and post-nasal cavity and chronic inflammation of the stomach and intestines; (2) that the invasion from the infected mouth and pharynx is supported by the fact that many of the known pathogenic micro-organisms present identical biological and morphological forms in cases of gastritis as the micro-organisms found in diseases of the mouths and post-nasal cavities of the same patients.

Freudenthal has shown by his experiments[25] that in catarrhal or obstructed conditions the mucous lining of the nose may give off as little as 57 grammes of watery vapour per day, instead of the 500 grammes yielded in a state of health, and consequently the inspired air reaches the pulmonary alveoli more or less dry and cold, and possibly not altogether germ free. He ascribes the frequency of post-nasal catarrh in America to the overheating of dwellings, the nasal mucosa in its endeavour to meet the necessity of moistening the dry, hot air becomes intumescent. Freudenthal utters a protest against too frequent or too free cauterisation of the nasal passages, and cites the case of a man who "was galvano-cauterised in his nose repeatedly, and is well since." Four years afterwards it was shown that the mucous membrane had been so destroyed that during one day it gave off only 167 grammes of water instead of 500.

Catarrhal rhinitis is not infective according to the results of bacteriological examination and the failure of inoculation experiments of Fernis and Bretschneider.[26] The authors hold that the nose suffers in such fevers as measles, not because it affords a ready passage of ingress for infective germs, but because it is a route of elimination for the products the disease.

**What Becomes of the Micro-organisms in the Inspired Air?**—It has been shown by St. Clair Thomson and R. T. Hewlett[27] that at least 1,500 microbes are inspired every hour in London, while it must be a common event for the number to reach 14,000. Yet it has been demonstrated by Strauss, Tyndall, and Gunning that expired air is practically germ-free; thus, in Strauss' experiments, of 609 micro-organisms inhaled only a single one was expired. Lister's observations on pneumothorax caused by a wound of the lung by a fractured rib seem to show that the organisms are arrested before they reach the alveoli, while the experiments of Hildebrandt indicate that this arrest takes place before the trachea is reached. Thomson and Hewlett's experiments confirm these observations, for they have examined the tracheal mucus from many recently-killed animals and have always found it sterile. If, then, the bacteria in inspired air are arrested in the nasal cavities, where does this take place? What becomes of them? And how are they got rid of? In a former paper the authors arrived at the conclusion that the mucous membrane of the normal nose seems to be usually quite free from micro-organisms, while the vestibules, vibrissæ and crusts are swarming with them. That the action of the ciliated epithelium is an important factor can hardly be doubted, for any particles on the dorsal wall of a frog's pharynx were seen to be moved along at the rate of twenty-five millimetres (one inch) per minute. By inoculating the nasal mucosa of the septum of one of the authors with culture of bacillus prodigiosus, it was shown that in eighty minutes the mucosa was almost free, while after two hours no growth of the bacillus could ever be detected. Proof was also obtained of non-bactericidal properties of nasal mucus, but it decidedly inhibited the growth of micro-organisms. Further proof of the fact that micro-organisms are caught in the nose is afforded by the experiment of testing the inspired air after its passage through the nose, when it was found that the microbes were practically all gone.

**Fibrinous Rhinitis.**—Recent observations on the pathology of fibrinous rhinitis are of the utmost importance and interest, not to the rhinologist only, but to every practitioner of medicine, for it has been shown that most, if not all, cases hitherto known clinically by the term fibrinous rhinitis are neither more nor less than diphtheria. The pathology of the affection was discussed at a meeting of the Incorporated Society of Medical Officers of Health[28] on March 19th. J. C. Heaven, in opening the discussion, related a series of facts which drew his attention to the subject. On November 20th, 1894, he was called to see a lady who had been rather suddenly seized with difficulty in swallowing, accompanied by some pain. There was apparently no throat affection, the fauces and tonsils presenting a normal appearance. She gradually improved, but was not quite well by December 17th, on which date L. H., aged four, and S. H., aged five and a-half, went to a party and were kissed by her. Two days after the boy L. H. had a slight nasal discharge from the right side, but remained otherwise in good health. Little attention was given to the matter till the 28th, when it was found that his nostril on this side was blocked, and by means of a director, under the influence of chloroform, a mass of whitish membrane an inch long and one-sixteenth of an inch thick was removed. The nostril was again becoming obstructed when on January 2nd his sister S. H. developed patches on the mucous membrane of the left nostril. Cultures from the nose of each child examined at the Bristol Public Health Laboratory showed Lœffler bacilli in considerable numbers. The presence and nature of these bacilli was confirmed by Klein, to whom material from the nose of each child was sent. He reported that there was no doubt about the diphtheria bacillus that it was virulent when injected into the guinea-pig and in morphological and cultural respects was typical. After two months' treatment the diphtheria bacillus was no longer present. During the whole of this period both children had been in their usual robust health, nor were there any other symptoms. Twelve weeks later, the mother, Mrs. H., who had become run down owing to previous influenza, complained of sore throat, &c., and in a few days developed faucial diphtheria. The cause of the infection was discovered when the children's noses were inspected, as crusts were found which contained the Lœffler bacillus. Not the least valuable part of Heaven's paper is his almost complete *résumé* of the literature of the subject, and he adduced sufficient evidence to convince most of his hearers of the correctness of his views as to the diphtheritic nature of fibrinous rhinitis.

**Hydrorhœa.**—Fink, of Hamburg, reports a case in in which the patient discharged in one hour forty grammes of clear watery fluid of sp. gr. 1,003.

[24] New York Med. Journ., Nov. 23, 1895. [25] Journ. of the Amer. Med. Ass., Nov. 9, 1895; Practitioner, Feb., 1896. [26] Arch. Lar. and Otol., Jan., 1896; Epit. Brit. Med. Journ., Feb. 8, 1896; [27] Lanc., Jan. 11, 1896; Journ. of Laryng., Mar., 1896. [28] Public Health, April, 1896.

